# Building for speech: designing the next-generation of social robots for audio interaction

**DOI:** 10.3389/frobt.2024.1356477

**Published:** 2025-01-03

**Authors:** Angus Addlesee, Ioannis Papaioannou

**Affiliations:** ^1^ Department of Mathematics and Computer Science, Heriot-Watt University, Edinburgh, United Kingdom; ^2^ Aveni AI, Edinburgh, United Kingdom

**Keywords:** social robots, spoken dialogue, accessibility, robotics, human-robot interaction, conversational AI

## Abstract

There have been significant advances in robotics, conversational AI, and spoken dialogue systems (SDSs) over the past few years, but we still do not find social robots in public spaces such as train stations, shopping malls, or hospital waiting rooms. In this paper, we argue that early-stage collaboration between robot designers and SDS researchers is crucial for creating social robots that can legitimately be used in real-world environments. We draw from our experiences running experiments with social robots, and the surrounding literature, to highlight recurring issues. Robots need better speakers, a greater number of high-quality microphones, quieter motors, and quieter fans to enable human-robot spoken interaction in the wild. If a robot was designed to meet these requirements, researchers could create SDSs that are more accessible, and able to handle multi-party conversations in populated environments. Robust robot joints are also needed to limit potential harm to older adults and other more vulnerable groups. We suggest practical steps towards future real-world deployments of conversational AI systems for human-robot interaction.

## 1 Introduction

**FIGURE 1:**
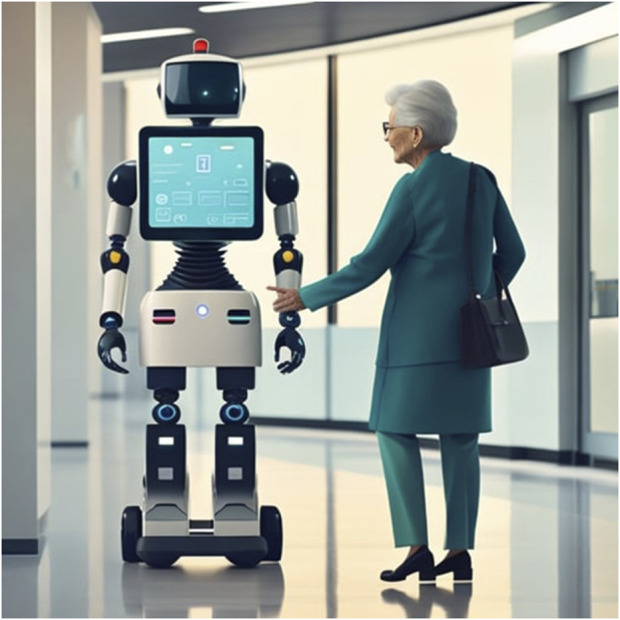
A person asking for directions in a hospital.

Social robots are not yet found in our public spaces, despite this vision being an imminent reality over 25 years ago (Thrun, 1998). They do not roam our shopping malls helping lost families find the bathroom, we do not bump into them providing departure times in train stations and airports, and they are not helping patients in hospital waiting rooms with their questions (see Figure 1).

Spoken dialogue systems (SDSs) have consistently improved over time (Glass, 1999; Williams, 2009; Lemon, 2022), with many years of peer-reviewed papers containing remarkable results. These models, however, are often evaluated automatically upon collected data, or with users in highly controlled lab settings. Social robots work wonderfully in the lab, but fail when deployed in the real world for experiments or demonstration (Tian and Oviatt, 2021). Some of these failures stem from the embedded SDS [for example: multi-party interactions (Addlesee et al., 2023a), socio-affective competence (Tian and Oviatt, 2021), voice accessibility (Addlesee, 2023), or trust failures (Tolmeijer et al., 2020)], but even interactions that the SDS should be able to handle with ease go wrong. These failures are often caused by the design of the social robot itself, classified by Honig and Oron-Gilad (2018) as technical hardware failures.

The field of robotics has also seen incredible advancements over the past years, today’s robots can navigate obstacle courses (Xiao et al., 2022), manipulate objects in their environment (Chai et al., 2022), generate human-like gestures (Tatarian et al., 2022), and follow complex human instructions using large language models (LLMs) (Ahn et al., 2022). Sadly, while collaboration between these two fields is common, they often begin after the robot has been designed. In our experience from multiple international robot dialogue projects, spoken interaction is not considered during the initial design phase. This lack of early-stage collaboration between robot designers and SDS researchers leaves room for oversight of critical features for spoken interaction, contributing to both performance and social errors (Tian and Oviatt, 2021). In this paper, we have combed the literature and drawn from our own experiences to highlight underlying and fundamental hardware problems that repeatedly surface when experimenting with social robots and real users. We hope this paper sparks discussion between both communities to create fully functioning social robots that do genuinely work in public spaces in the future, enabling live in-the-wild experiments.

## 2 People struggle to hear robots

The first issue that crops up commonly in the literature is the limited volume of the robot’s voice. Robot designers simply attach a speaker to the robot without considering the fact that the world is noisy, and some users, such as older adults, may have hearing loss.

In recent work, researchers deployed a robot to interact with real users in an assisted living facility. The robot had to be fitted with an additional speaker that had a louder maximum volume. The modification was necessary because the users simply could not hear the robot’s voice, preventing any basic interaction (Stegner et al., 2023).

This issue is not constrained to this particular setting, or to one particular robot. For example, researchers had to repeat every sentence the robot said in the lobby of a concert hall, as participants could not hear it Langedijk et al. (2020). In various school environments, the robot’s volume was not loud enough to enable effective interaction, so external speakers had to be fitted (Nikolopoulos et al., 2011). When guiding people in an elder care facility, the robot’s single speaker faced the wrong direction, so users could not hear it Langedijk et al. (2020). Another robot was deployed in the homes of a few older adults, and they noted that its volume was not loud enough. People could not hear a social robot in a gym (Sackl et al., 2022), and the list goes on.

Robots are expensive, but the speakers that researchers had to retrofit to the robots were inexpensive and readily available. This low-cost change was simple, yet *crucial*, to enable effective communication with a user in a real-world setting. When designing robots for spoken interaction, we recommend fitting multiple speakers (facing various directions) that have a loud maximum volume. This will guarantee that the robot can be heard in public spaces, and ensure its accessibility for people with limited hearing. In the future, parametric array loudspeakers (PALs) could be installed to use ultrasonic transducers (Yang et al., 2005; Bhorge et al., 2023). PALs are unidirectional, using the nonlinear interactions between soundwaves to enable directed personal communication to a specific user in a populated environment (Zhu et al., 2023).

## 3 Robots struggle to hear people

There is another conversation participant that cannot properly hear what their interlocutor is saying–the robot. This problem is similar to the one in Section 2, and is also frequently found in the literature. A social robot struggled to hear users in a hotel lobby, for example (Hahkio, 2020). Many researchers retrofit better microphones to the robot (Villalpando et al., 2018), or next to the robot (Wagner et al., 2023), in order to hear the user more clearly.

In an assisted living facility, researchers had to resort to listening to the user through an ajar door to run their experiments. The microphone array could not reliably pick up what users said (Stegner et al., 2023).

Home voice assistants do successfully hear people in noisy environments, like family homes (Porcheron et al., 2018), however. They can pick up what the user said when other conversations are happening in the room, and when the TV or radio are on [we are also finding this in ongoing work (Addlesee, 2022)]. Today’s social robots typically have four microphones^1^, but we argue that this is far too few. Apple’s Homepod originally had six microphones (Calore, 2019), and Amazon’s Alexa Echo had seven (Spekking, 2021). The newest Homepod and Echo have reduced to four microphones for two reasons: (1) These devices are incentivised to keep their device’s costs low to encourage adoption by new users (Welch, 2023); and (2) The device’s shape and internal component arrangements have been refined and optimised over many years through experiments with millions of users (Wilson, 2020). Robots do not share either of these features. Microphones are trivially inexpensive relative to the price of a robot, and instead of helping microphones, the robot’s shape actively hinders their performance. The body parts of a social robot often sit between the user and the microphone (for example, when the user is behind the robot, or in a wheelchair). Robots also create a lot of noise themselves, called *ego-noise*. Related research required high-quality audio input from a noisy propellered UAV, so they attached sixteen microphones in various locations around the device (Nakadai et al., 2017), not just four.

Human-robot spoken communication can also be disrupted by societal or linguistic phenomena, such as overlapping or poorly formed turn-taking conditions (Skantze, 2021). Such conditions include barging-in (Wagner et al., 2021) (where the user interrupts the robot mid-sentence, but the robot fails to recognise that the user started speaking), and poor end-of-turn detection (due to long pauses or intermittent speech from the user). In our experience, users sometimes barge-in because of high latency caused by limited computational power onboard the robot, or on-site connectivity issues, in addition to the SDS latency.

Potential approaches to this challenge include incremental dialogue processing (Addlesee et al., 2020; Aylett et al., 2023), predictive turn-taking (Inoue et al., 2024), or explicit turn-taking signals which enable the user to better understand when the robot is actually listening to them. For instance, Foster et al. (2019) employed a tablet on the robot’s torso that was showing *“I am listening”* and *“I am speaking”* text to help guide the users in a noisy shopping mall setup.

We therefore recommend fitting multiple high-quality microphones in various locations around the robot’s body, as well as using appropriate signal processing techniques, such as beam-forming (Adel et al., 2012). Latency issues must be addressed within the SDS, and by increasing the robot’s computational capabilities. These changes will again ensure that multi-party spoken interactions can realistically take place in public spaces. Robot designers must also consider microphone placement lower down on the robot for shorter users, and for people in wheelchairs, as they are commonly just placed on the top of the robot’s head.

### 3.1 Multi-party interaction

All of the above challenges assume that the interaction is dyadic–that is, one person conversing with a single system/robot. Conversational AI systems and SDSs are typically designed for this setting, including commercial assistants like Alexa and Siri. However, dyadic interactions can only be guaranteed in specific environments, such as single-occupant homes (and even then, there may be visitors). In the public spaces that social robots are expected to roam in the future (see Section 1), groups of people may approach the robot (Alameda-Pineda et al., 2024). In multi-party conversations (MPCs), the SDS must track who said an utterance, who the user was addressing, and then generate a suitable response, depending on whether the robot is addressing an individual or the whole group (Traum, 2004). The robot may also need to decide to remain silent, for example if people are talking to each other, but still monitor the content of their conversation in case it can assist them. Additionally, MPCs introduce unique challenges such as multi-party goal-tracking (Addlesee et al., 2023b). Groups may have conflicting goals, or share goals (Eshghi and Healey, 2016). Current social robots are not designed to enable MPCs, since speaker diarization (tracking ‘who said what’) is critical (Addlesee et al., 2023c; Schauer et al., 2023). The audio from the robot’s microphones must not only be clear enough to perform ASR accurately, but clear enough to determine *who* said an utterance (Cooper et al., 2023). Ideally, the microphones would also provide the angle which the audio originated from. This angle can be combined with the robot’s vision to determine which person in view said an utterance. The robot can then look at the user it is addressing when responding.

We recommend that social robots be designed with multi-party interaction in mind–this means designing microphone arrays such that speaker diarization is accurate, combining this with person-tracking, and developing NLP systems that can understand and manage multi-party conversations (Lemon, 2022).

## 4 Ego-noise

This issue of ego-noise, introduced in Section 3, is so problematic that an entire field of research has grown to tackle it. Researchers find that ego-noise, noise generated by the robot itself, does not just negatively impact ASR performance, but that ego-noise reduction methods also suppress some of the user’s utterance (Ince et al., 2010; Schmidt et al., 2018). To clarify, both the ego-noise reduction techniques, and the ego-noise itself negatively impact ASR performance (Alameda-Pineda et al., 2024).

This issue would be helped by additional speakers and microphones, ideally not placed next to noise sources, allowing both parties to hear each other. An optimal social robot designed for spoken interaction would also have much quieter joint motors and fans. These are more expensive than speakers and microphones, but they would greatly improve the SDSs ability to understand the user. This could be paired with research to repair and understand disrupted sentences (Addlesee and Damonte, 2023a; Addlesee and Damonte, 2023b), while quieter motors are developed.

In addition to joint motors and fans, the robot’s own voice is another source of ego-noise. Microphones cannot simply be turned off when the robot is talking, as speech can be overlapping, so recognised speech may have to be classified as being produced by itself or another (Lemon and Gruenstein, 2004).

## 5 Joint robustness

**FIGURE 2:**
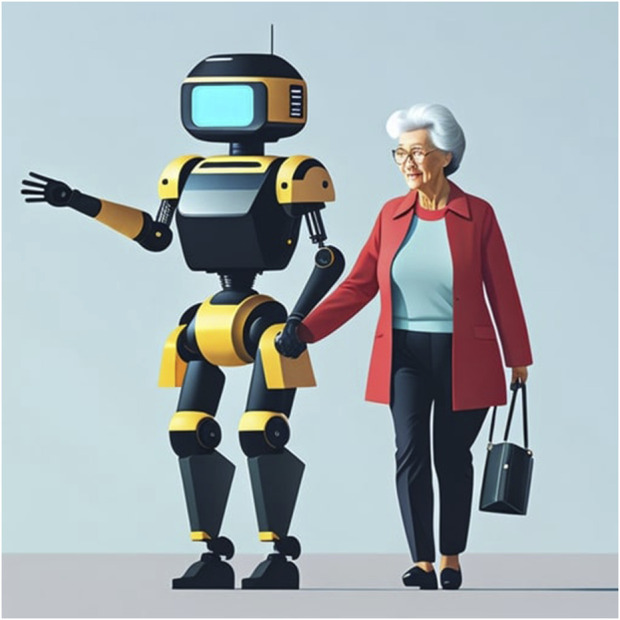
A social robot providing stability to an older adult.

Ego noise obviously does not impact robots that do not have a body. In our view, though, social robots should be able to point to location and objects, guide users, and help users physically. For example, consider a hospital waiting room in a hospital memory clinic (Gunson et al., 2022). Patients are typically older adults, and may use the robot’s arm for stability, like they would with another human (see Figure 2). Current social robots can generate social gestures like waving or holding its hand out for a handshake. If you were to shake the robot’s hand, however, it would likely break. Such fragility could potentially harm users if deployed in this setting. People may assume that they can link arms with the robot while being guided, a perfectly natural assumption. When an older adult puts their weight on the robot’s joint, though, they might fall. This is clearly a potentially harmful design flaw that must be resolved if we are ever going to find robot assistants in the wild.

## 6 Conclusion

Interacting naturally with social robots in public spaces is currently still a sci-fi fantasy. There are challenges that SDS researchers must tackle to reach this goal, but that is not the only bottleneck. Even a perfect SDS would fail if it was embedded within today’s social robots. We have highlighted that robots need louder speakers (or parametric array loudspeakers in the future), a greater number of high-quality microphones, quieter fans, and quieter motors to allow both parties to hear each other. These are critical problems that completely block spoken interactions outside a lab setting. We highlighted that robots also need to be more physically robust if they are to be safely applied in the real world, particularly in settings with older adults.

Social robotics research will continue to rely on offline evaluations, wizard-of-oz deployments, or lab-based experiments if these robot hardware issues are not resolved. Our suggestions are not an exhaustive list, but we hope that they spark discussion and encourage collaboration between robot designers and SDS researchers. This collaboration should take place in the initial stages of a robot’s design to avoid the retrofitting of hardware and sensors discussed in this paper, and instead enable real in-the-wild experiments.

## Data Availability

The original contributions presented in the study are included in the article/supplementary material, further inquiries can be directed to the corresponding author.

## Footnotes

1 We checked the technical specifications of several commonly used social robots, and robots that we have deployed ourselves. We are refraining from naming specific robot creators, as this paper aims to encourage collaboration, and not criticise specific robots.
